# miR-126 overexpression attenuates oxygen-glucose deprivation/reperfusion injury by inhibiting oxidative stress and inflammatory response via the activation of SIRT1/Nrf2 signaling pathway in human umbilical vein endothelial cells

**DOI:** 10.3892/mmr.2021.12158

**Published:** 2021-05-18

**Authors:** Jixin Li, Caili Yang, Yan Wang

Mol Med Rep 23: Article no. 165, 2021; DOI: 10.3892/mmr.2020.11804

Following the publication of this article, the authors have realized that the two affiliation addresses in the paper were written incorrectly: “The First People's Hospital” and “The Second People's Hospital” should have appeared as “The First People's Hospital **of Linhai**” and “The Second People's Hospital **of Linhai**”, respectively. Therefore, the author affiliations and addresses in this paper should have appeared as follows:

JIXIN LI^1^, CAILI YANG^1^ and YAN WANG^2^

^1^Department of Neurology, The First People's Hospital of Linhai, Taizhou, Zhejiang 317000; ^2^Department of Neurology, The Second People's Hospital of Linhai, Taizhou, Zhejiang 317016, P.R. China

The authors regret these errors in the presentation of the affiliation addresses, and apologize for any inconvenience caused.

